# A phase 1 open label study to assess the human mass balance and metabolite profile of ^14^C-fosmanogepix, a novel Gwt-1 inhibitor in healthy male participants

**DOI:** 10.1128/aac.00273-24

**Published:** 2024-07-16

**Authors:** Michael R. Hodges, Eric Ople, Philip Evans, A. J. (Andre) Pantophlet, Jessica Richardson, Dylan Williams, Sakambari Tripathy, Margaret Tawadrous, Abhijeet Jakate

**Affiliations:** 1 Independent/Former Amplyx and Pfizer, San Diego, California, USA; 2 Amplyx Pharmaceuticals, Inc., San Diego, California, USA; 3 Quotient Sciences, Ruddington Fields, Nottingham, United Kingdom; 4 PRA Health Sciences (now ICON PLC) - ICON Bioanalytical Laboratories, Assen, the Netherlands; 5 Pharmaron UK Ltd., Rushden, Northamptonshire, United Kingdom; 6 Pfizer Inc., New York, New York, USA; University of Iowa, Iowa City, Iowa, USA

**Keywords:** carbon-14 (^14^C), FMGX/APX001, MGX/APX001A, radioactivity, metabolites

## Abstract

Fosmanogepix [FMGX; active form manogepix (MGX)], a novel antifungal, is currently being studied for the treatment of invasive fungal diseases caused by *Candida* spp., *Aspergillus* spp., and other rare molds. This Phase 1, single-dose study used ^14^C-radiolabeled FMGX to determine the disposition and metabolism of FMGX. Ten healthy male participants were enrolled equally into: oral cohort {FMGX 500 mg oral + 3.1 megabecquerel [MBq, 84.0 microcurie (μCi)] ^14^C} and intravenous (IV) cohort [FMGX 600 mg IV + 3.4 MBq (93.0 µCi) ^14^C]. At the end of the sampling period (456 h post-dose), 90.2% of radioactivity administered was recovered (46.4% from urine; 43.8% from feces) in oral cohort (82.3% within 240 h), and 82.4% was recovered (42.5% from urine; 39.9% from feces) in IV cohort (76.2% within 264 h), indicating that FMGX elimination occurs via renal and hepatic routes. Radioactivity transformation pathways (oral and IV) indicated multiple major routes of metabolism of FMGX, mainly via MGX, and included oxidation, oxidative deamination, and conjugation. All except one key human plasma metabolite was observed in toxicity species, but its proportion (<10%) in the human area under the curve plasma samples was not of toxicological concern. No deaths, serious, or severe adverse events (AE) were reported, and there were no AE-related withdrawals. The results of this study indicated extensive metabolism of FMGX, with similar key human plasma metabolites observed in the animal studies. The elimination of FMGX was equally through renal and hepatic routes.

**CLINICAL TRIALS:**

This study is registered with ClinicalTrials.gov as NCT04804059.

## INTRODUCTION

Globally, invasive fungal diseases (IFDs) affect ≥3 million people (1). An increase in the occurrence of IFDs due to an increase in the number of immunocompromised patients has led to significant mortality, morbidity, and a rise in healthcare costs (1, 2). Currently, available antifungal agents (polyenes, echinocandins, and azoles) may have limited use due to toxicity, narrow spectrum of activity, limited routes of administration, drug-interaction liabilities, emerging resistance, etc., necessitating the development of alternative and effective antifungal therapies (3).

Fosmanogepix (FMGX) is the first member of the “gepix” class of antifungals with a novel mechanism of action and is currently under clinical development. It is a water-soluble prodrug that is rapidly and completely metabolized *in vivo* by systemic alkaline phosphatases to the active moiety, manogepix (MGX) (4, 5). MGX acts by inhibiting the fungal glycosylphosphatidylinositol (GPI)-anchored wall protein transfer 1 (Gwt-1), a gene that encodes acyltransferase involved in GPI-anchored mannoprotein biosynthesis, an important component of the fungal cell wall responsible for fungal adhesion and pathogenicity (6). Inhibition of Gwt-1 compromises cell wall integrity, restricts fungal growth, and prevents fungal invasion into host cells (7, 8).

MGX has shown potent *in vitro* activity against most *Candida* species (including fluconazole- and echinocandin-resistant species), *Aspergillus* spp., *Fusarium* spp., *Scedosporium* spp., etc. In animal models, MGX has also demonstrated activity against multiple yeasts (*Candida* and *Cryptococcus* spp.) and mold pathogens (*Aspergillus* spp. and rare molds) (6, 8). In Phase 1 studies in healthy volunteers, single and multiple intravenous (IV; up to 1,500 mg) and oral doses of FMGX (up to 1,000 mg) were evaluated. FMGX was well tolerated at doses of up to 1,000 mg IV and 800 mg oral and only mild, transient adverse events (AEs) that did not require intervention were reported. In both Phase 1 studies, MGX demonstrated a linear and dose-proportional pharmacokinetic (PK) profile, high oral bioavailability (90.6%–101.2%), and no effect of food was observed on MGX exposures (4). Although these data describe the FMGX and MGX PK profiles, other details of FMGX metabolism in the human body, including mass balance, routes of elimination, and metabolic profile have not been reported. Mass balance studies, using radioactive labeling, elucidate the disposition and metabolism of a drug and are an important part of the clinical pharmacology findings submitted to regulatory authorities.

Previously, the absorption, distribution, and excretion profiles of carbon-14 (^14^C)-FMGX were studied in rats (single 100 mg/kg oral or 30 mg/kg IV dose) and monkeys (single 6 mg/kg IV dose). ^14^C-FMGX was found to be rapidly and extensively absorbed and distributed to most tissues via both oral and IV routes in both rats and monkeys and was excreted primarily by the biliary/fecal route (9). This Phase 1, open-label, single-dose study was conducted to assess the mass balance recovery, metabolite profile, and identification of ^14^C-FMGX in healthy male participants after oral and IV dosing. This study will help in identifying metabolites of FMGX, understanding primary routes of excretion, and the extent of distribution into blood cells in humans (10, 11).

## MATERIALS AND METHODS

### Study design and participants

In this single-center, non-randomized, single-dose, Phase 1 study (NCT04804059) (12), 10 healthy male participants aged 30–65 years, with a body mass index between 18.0 and 32.0 kg/m^2^, regular bowel movements (average stool production 1–3 times per day), and assessed to be in general good state of health were included. Key exclusion criteria included administration of any investigational drug in a clinical research study (within the last 3 months) or a ^14^C radioactive clinical trial (within the last 1 year); radioactive exposure (including exposure from the present study, diagnostic x-rays, and other medical exposures) exceeding 5 millisieverts (mSv) in the last 1 year or 10 mSv in the last 5 years; occupationally exposed workers (per Ionizing Radiation Regulations 2017); renal impairment (creatinine clearance <80 mL/min using Cockcroft-Gault equation); administration of any prescribed/over-the-counter drug (except 4 g/day paracetamol)/herbal remedies 14 days before drug administration. Participants were also excluded if they had tested positive for hepatitis B surface antigen, hepatitis C virus antibody, or HIV or had a history of clinically significant cardiovascular, renal, hepatic, chronic respiratory or gastrointestinal disease, neurological, or psychiatric disorder [as judged by the principal investigator (PI)].

### FMGX administration

**Fig 1:**
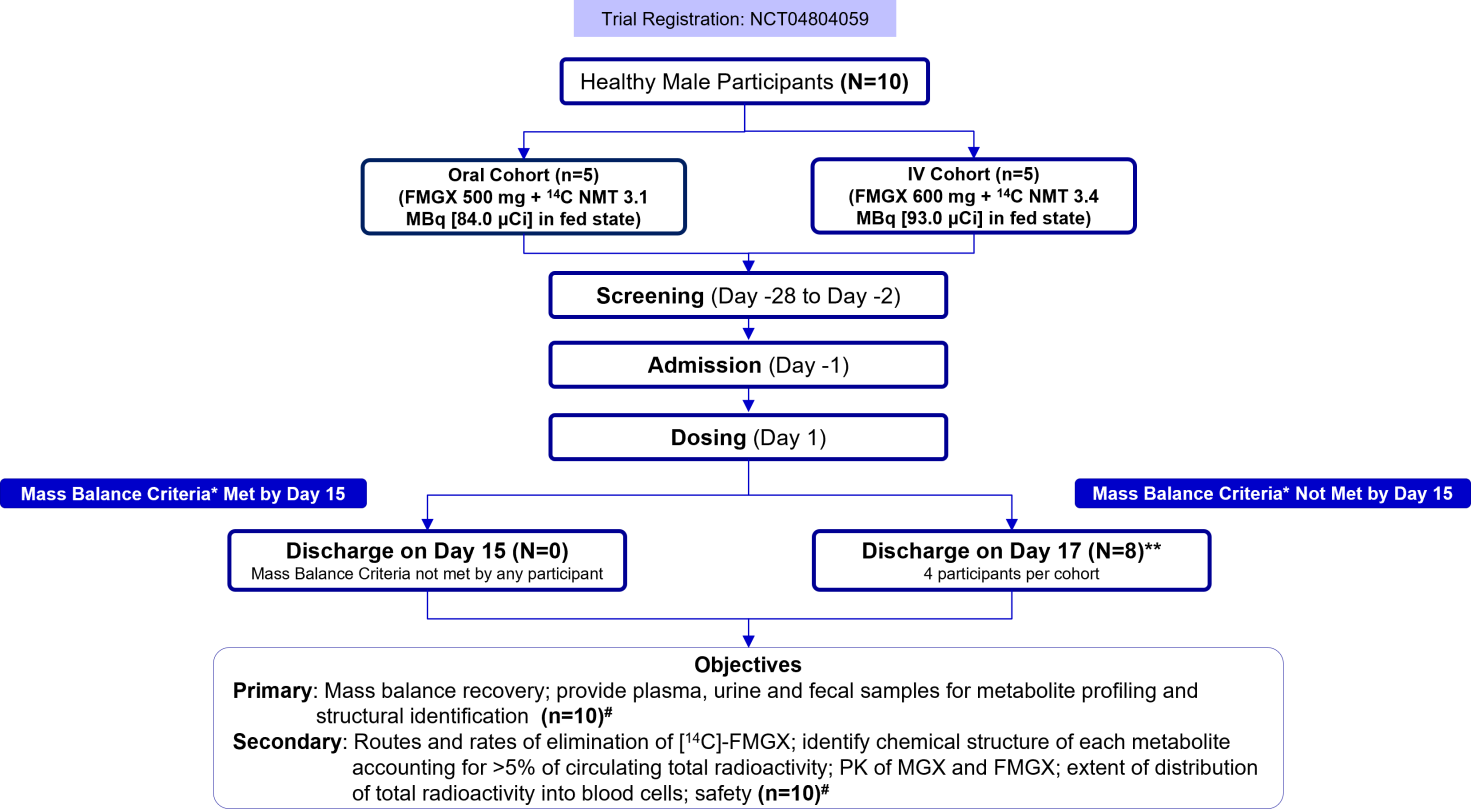
Study design. *Mass balance cumulative recovery of >90% or if <1% of the dose administered had been collected in urine and feces within two separate, consecutive 24-h periods. If participants were in the unit until day 17, all safety assessments scheduled for day 15 were to take place on day 17. Urine and feces collection continued until discharge, but plasma and whole blood samples for mass balance, PK, and metabolite profiling and identification assessments were only taken until day 15. If the criteria were not met by day 17, or additional residency was not considered appropriate or necessary, home collections of urine and/or feces may have been requested at the discretion of the PI for individual participants. **All participants were resident in the unit until day 17, with the exception of one participant in the oral cohort (left on day 11) and one participant in IV cohort (left on day 15) due to reasons other than meeting mass balance criteria; #home collections of urine and feces were required from all subjects, up to 456 h post-dose (day 20). ^14^C, carbon-14; μCi, microcurie; FMGX, fosmanogepix; IV, intravenous; MBq, megabecquerel; MGX, manogepix; NMT, not more than; PK, pharmacokinetics.

A total of 10 participants were enrolled into oral or IV cohorts (five participants each). Participants in the oral cohort received a single oral dose of FMGX 500 mg and not more than (NMT) 3.1 megabecquerel [MBq, 84.0 microcurie (μCi)] ^14^C in the fed state. Participants in the IV cohort received a single IV dose of FMGX 600 mg (via a 3-h infusion) and NMT 3.4 MBq (93.0 µCi) ^14^C in the fed state. After a screening period of 28 days (day −28 to day −2), participants were admitted to the clinical unit on day −1, a day before FMGX dosing (day 1), and were to remain in the unit for up to 384 h after dosing (up to day 17). It was planned that all participants in the cohort would be released if they achieved a mass balance cumulative recovery of >90% or if <1% of the dose administered had been collected in urine and feces within two separate, consecutive 24-h periods. If mass balance criteria were not met by all participants by day 15, the residency period was extended by a maximum of 48 h (day 17). If these criteria were not met by day 17, the collection of urine and feces could continue through either additional residency or home collections of urine and/or feces (per PI decision; Fig. 1).

### Assessments

The primary objectives of this study were to assess the mass balance recovery after a single oral or IV dose of ^14^C-FMGX, and metabolite profiling, and structural identification. The secondary objectives were to determine the routes and rates of elimination, identify the chemical structure of each metabolite accounting for >2% of the circulating radioactivity in plasma and >5% of the dose in urine and feces, further explore FMGX and MGX PK, and evaluate the extent of distribution of total radioactivity into blood cells and safety. In both cohorts, plasma and whole-blood samples for total radioactivity (mass balance), PK, and metabolite profiling and identification were collected at specified time points from day 1 to day 15. Urine and fecal samples were, however, collected until discharge (day 17) or at home as stated above. Safety was assessed by the frequency of AEs recorded from the day of FMGX dosing (day 1) until discharge (day 17) in both cohorts. All AEs were classified per the Medical Dictionary for Regulatory Activities (MedDRA v20.1) and ascertained as related to study drug administration or not by the PI.

The total radioactivity concentrations in plasma, whole blood, urine, and feces were determined by liquid scintillation counting, and plasma FMGX and MGX PK concentrations were determined using liquid chromatography with tandem mass spectrometry (LC-MS/MS). For mass balance analysis, cumulative amounts of total radioactivity excreted (CumAe) in urine, feces, and total (urine and feces combined) were calculated along with cumulative recovery (Cum%Ae) estimated as CumAe expressed as a percentage of the radioactive dose administered. For PK analysis, in both cohorts, the following plasma parameters were estimated for MGX and total radioactivity: maximum observed concentration (C_max_); time of C_max_ (T_max_); area under the curve (AUC) from 0 time to 24 h post-dose [AUC_(0–24)_]; AUC from 0 time to the last measurable concentration [AUC_(0–last)_]; AUC from 0 time extrapolated to infinity [AUC_(0–inf)_]; apparent elimination half-life (T_1/2_); total body clearance (CL); apparent volume of distribution based on the terminal phase (V_d_); mean residence time from 0 time to the last measurable concentration [MRT_(0–last)_] and extrapolated to infinity [MRT_(0–inf)_], both post extravascular and intravascular administration for the oral and IV cohorts, respectively; C_max_, AUC_(0–last)_, and AUC_(0–inf)_ divided by actual dose D (i.e., C_max_/D, AUC_(0–last)_/D, and AUC_(0–inf)_/D, respectively). Absolute bioavailability (F) was calculated as a ratio of geometric mean (GM) C_max_/D, AUC_(0–last)_/D, and AUC_(0-inf)_/D after oral dose divided by C_max_/D, AUC_(0–last)_/D, and AUC_(0–inf)_/D after IV dose, respectively. Whole blood to plasma total radioactivity ratios were calculated at relevant whole-blood time points. In addition, metabolite-to-parent ratios (MPRs) based on C_max_ (MPR C_max_) and AUC_(0–last)_ [MPR AUC_(0–last)_] were reported only in the IV cohort. FMGX PK parameters [T_max_, C_max_, and AUC_(0–last)_] were estimated only in the IV cohort. The plasma PK parameters for MGX, total radioactivity, and FMGX were estimated using Phoenix WinNonlin software (v8.0, Certara Inc., USA).

### Metabolite profiling and structural identification

Plasma, urine, and fecal samples were analyzed using high-resolution LC-MS/MS with in-line fraction collection and off-line counting to obtain ^14^C-radiochromatographic profiles and provide information on the nature of the radioactive components present. Structural identification was attempted for all radioactive components representing >2% circulating radioactivity in plasma or representing >5% of the dose in urine and feces. In addition, pooled rat, mini-pig, and monkey plasma samples were analyzed alongside matrix-matched human plasma samples to determine the presence/absence of the key human plasma metabolites and perform semi-quantification of the metabolites in the animal samples.

### Sample size and analysis sets

In this exploratory study, no formal sample size calculation was conducted. A total of 10 participants were enrolled (5 per cohort), with a target of including 8 evaluable participants (4 per cohort), which was considered sufficient for analysis. The safety analysis population included all participants who received at least one dose of FMGX. These participants were included in the PK analysis population if they had a minimum of one valid post-dose analytical result for PK parameter estimation, had no missing samples at critical time points, e.g., around C_max_, and no relevant protocol deviations or AEs (e.g., vomiting) suggesting low/no absorption of a whole dose (in the oral cohort). The mass balance population included all participants who received at least one dose of FMGX and had evaluable total radioactivity concentration (urinary and fecal) data without protocol deviations (e.g., spillage of urine and/or feces; missing collections) or AEs suggesting low/no dose absorption. No formal statistical analysis was required for the mass balance and PK data, and no interim statistical analysis was planned for this study.

## RESULTS

### Participant disposition and demographics

All 10 participants enrolled (five each in the oral and IV cohorts) received ^14^C-FMGX, completed the study, and were included in the safety, PK, and mass balance populations. The mass balance criteria were not met by any participant by day 15, and home collections of urine and feces were required from all participants, up to 456 h post-dose (day 20). Participants in both cohorts had comparable demographic characteristics with the majority white males aged [mean (SD)] 48.8 years (9.4) and 40.2 years (9.1) in the oral and IV cohorts, respectively (Table 1).

**TABLE 1:** Baseline and demographic characteristics (safety population)^*a*^

Baseline characteristics	Oral cohort [FMGX 500 mg + ^14^C NMT 3.1 MBq (84.0 µCi)] *n* = 5	IV cohort [FMGX 600 mg + ^14^C NMT 3.4 MBq (93.0 µCi)] *n* = 5
Age (yr), mean (SD)	48.8 (9.4)	40.2 (9.1)
Gender, *n* (%)		
Male	5 (100)	5 (100)
Race, *n* (%)		
White	5 (100)	4 (80)
Other	0	1 (20)
BMI (kg/m^2^), mean (SD)	25.5 (3.21)	27.8 (3.33)

^*a*14^ C, carbon-14; μCi, microcurie; BMI, body mass index; FMGX, fosmanogepix; IV, intravenous; MBq, megabecquerel; NMT, not more than; SD, standard deviation; yr, years.

### Mass balance analysis

**Fig 2:**
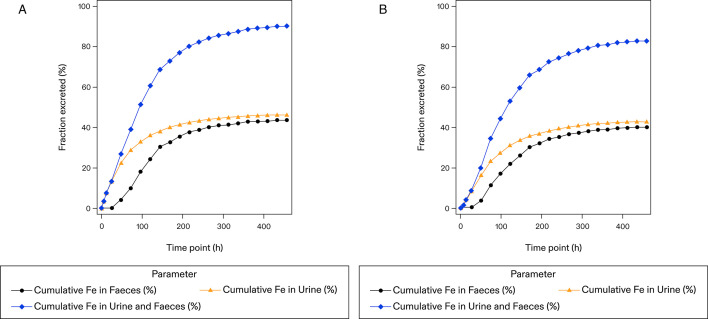
Arithmetic mean ± SD cumulative recovery (%) of total radioactivity: linear/linear scale (mass balance population). (**A**) Oral cohort: FMGX 500 mg + ^14^C NMT 3.1 MBq (84.0 µCi). (**B**) IV cohort: FMGX 600 mg + ^14^C NMT 3.4 MBq (93.0 µCi) ^14^C, carbon-14; μCi, microcurie; Fe, fraction of total radioactivity recovered in urine, feces, and total (urine and feces combined); FMGX, fosmanogepix; h, hour; IV, intravenous; MBq, megabecquerel; NMT, not more than.

At the end of the sampling period (456 h post-dose/day 20), in the oral cohort, a mean of 90.2% of the radioactivity administered was recovered, including 46.4% recovery from urine and 43.8% from feces. In the IV cohort, a mean of 82.4% of the radioactivity administered was recovered, including 42.5% recovery from urine and 39.9% from feces (Table 2). Within the first 24 h post-dose, the total radioactivity recovered in urine and feces was 13.2% and 0.25%, respectively, in the oral cohort and 8.06% and 0.29%, respectively, in the IV cohort (Fig. 2). Additionally, of the total radioactivity recovered in both cohorts (90.2%: oral cohort and 82.4%: IV cohort), about 90% (82.3%: oral cohort and 76.2%: IV cohort) was obtained by 240 h in the oral cohort and by 264 h in the IV cohort.

**TABLE 2:** Mass balance recovery in urine and feces (mass balance population) and summary of plasma PK parameters for total radioactivity (PK Population)^*e*^

	Oral cohort [FMGX 500 mg + ^14^C NMT 3.1 MBq (84.0 µCi)] *n* = 5	IV cohort [FMGX 600 mg + ^14^C NMT 3.4 MBq (93.0 µCi)] *n* = 5
Mass balance recovery parameters^*a*^, mean (SD)		
Mean CumAe (total, mg equiv)		
Urine	232 (33.7)	270 (56.6)
Feces	219 (19.1)	252 (23.9)
Total	451 (30.9)	522 (53.4)
Mean CumAe %		
Urine	46.4 (6.73)	42.5 (8.72)
Feces	43.8 (3.81)	39.9 (4.01)
Total	90.2 (6.18)	82.4 (8.33)
Total radioactivity plasma PK parameters^*b*^		
T_max_ (h)	3.00 (1.00–4.06)	3.01 (3.00–3.07)
C_max_ (ng equiv/mL)	9740 (22.1)	12,300 (6.80)
C_max_/D (ng equiv/mL/mg)	19.5 (22.2)	19.4 (7.80)
AUC_(0-24)_ (ng equiv.h/mL)	122,000 (29.1)	129,000 (12.8)
AUC_(0-last)_ (ng equiv.h/mL)	410,000 (18.5)	562,000 (23.1)
AUC_(0-last)_/D (ng equiv.h/mL/mg)	820 (18.5)	887 (21.5)
AUC_(0-inf)_ (ng equiv.h/mL)	446,000 (17.2)	603,000 (23.9)
AUC_(0-inf)_/D (ng equiv.h/mL/mg)	892 (17.2)	951 (22.3)
T_1/2_ (h)	61.6 (59.9)	69.5 (42.7)
CL^*c*^ (mL/min)	18.7 (17.2)	17.5 (22.3)
V_d_^*c*^ (L)	99.6 (53.9)	105 (24.7)
V_ss_ (L)	–^*f*^	102 (24.0)
MRT_(0-last__)_^*d*^ (h)	66.6 (47.5)	78.1 (41.5)
MRT_(0-inf)_^*d*^ (h)	86.9 (56.4)	97.1 (43.4)

^*a*^ At end of sampling period (0–456 h).

^*b*^ Reported as geometric mean (geometric coefficient of variation%) except for T_max_, which are reported as median (range).

^*c*^ CL/F and Vd/F for oral cohort.

^*d*^ Include MRT_(0–inf)__ev and MRT_(0–last)__ev for oral cohort and MRT_(0–inf)__iv and MRT_(0–last)__iv for IV cohort.

^*e*^ ^14^C, carbon-14; μCi, microcurie; AUC_(0–24)_: area under the curve (AUC) from 0 time to 24 h post-dose; AUC_(0–inf)_/D: AUC from 0 time extrapolated to infinity divided by actual dose; AUC_(0–inf)_: AUC from 0 time extrapolated to infinity; AUC_(0–last)_/D: AUC from 0 time to the last measurable concentration divided by actual dose; AUC_(0–last)_: AUC from 0 time to the last measurable concentration; CL: total body clearance after IV administration; C_max_/D: maximum observed concentration divided by actual dose; C_max_: maximum observed concentration; CumAe (total), cumulative amount of total radioactivity excreted in urine, feces, and total (urine and feces combined); CumAe %, cumulative amount of total radioactivity excreted in urine, feces, and total (urine and feces combined) expressed as a percentage of the radioactive dose administered; FMGX, fosmanogepix; h, hour; IV, intravenous; MBq, megabecquerel; MRT_(0–inf)__ev: mean residence time (MRT) extrapolated to infinity after extravascular administration; MRT_(0–inf)__iv: MRT extrapolated to infinity after IV administration; MRT_(0–last)__ev: MRT from 0 time to the last measurable concentration after extravascular administration; MRT_(0–last)__iv: MRT from 0 time to the last measurable concentration after IV administration; NMT, not more than; PK, pharmacokinetics; T_1/2_: apparent elimination half-life; CL/F: total body clearance after extravascular administration; SD, standard deviation; T_max_: time of maximum observed concentration; Vd/F: apparent volume of distribution based on the terminal phase after extravascular administration; V_d_: apparent volume of distribution based on the terminal phase after IV administration; V_ss_: predicted volume of distribution at steady state after IV administration.

^*f*^ –, not calculated.

### PK analysis: total radioactivity

**Fig 3:**
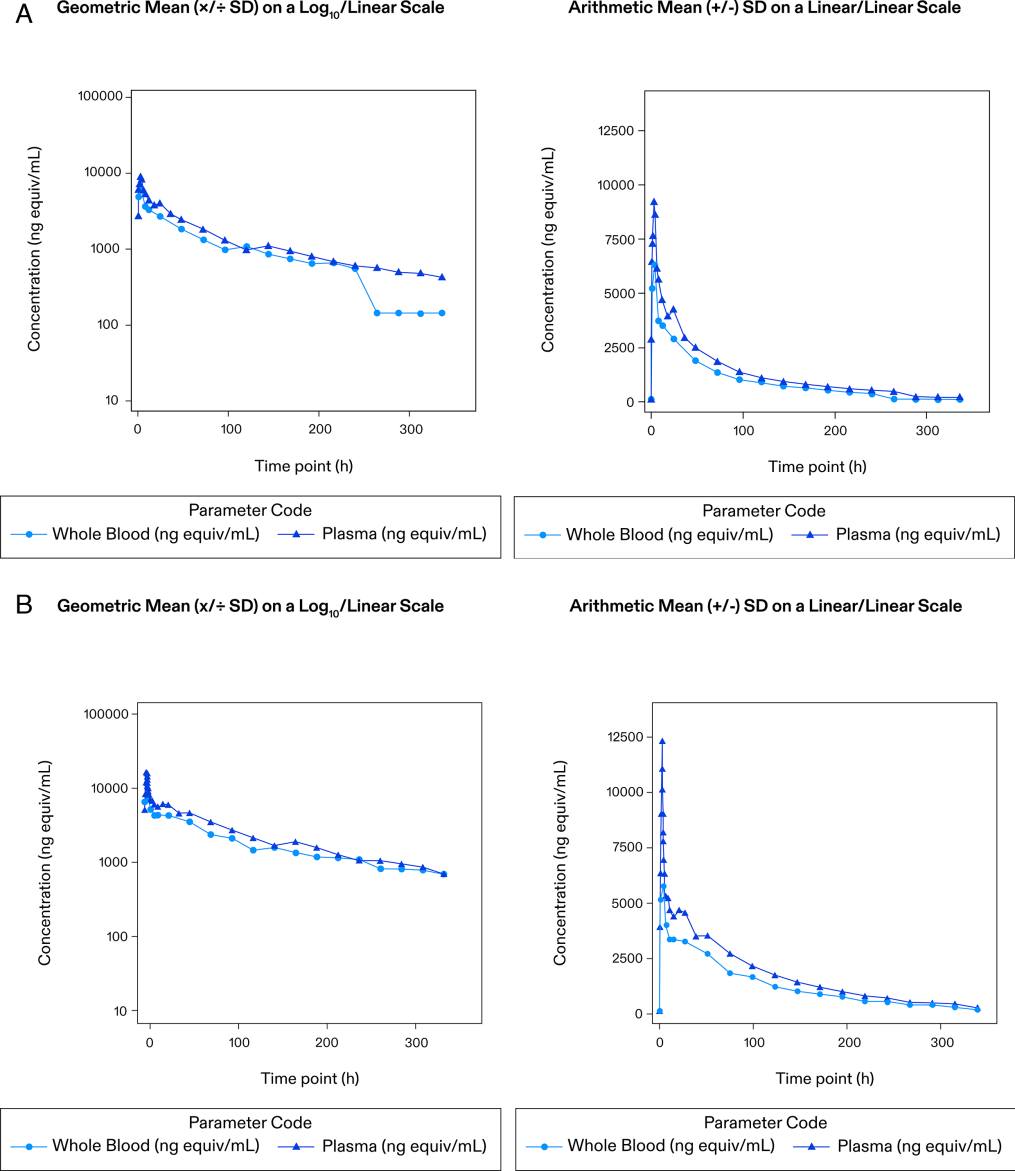
(**A**) Whole blood and plasma PK total radioactivity concentrations (PK population)-oral cohort. ^14^C, carbon-14; μCi, microcurie; FMGX, fosmanogepix; h, hour; IV, intravenous; MBq, megabecquerel; NMT, not more than; PK, pharmacokinetics; SD, standard deviation. (**B**) Whole blood and plasma PK total radioactivity concentrations (PK population)-IV cohort.

In both cohorts, plasma and whole-blood total radioactivity concentrations were observed from 0.50 h and 1.00 h post dose, respectively, in all participants. Maximum plasma concentrations occurred between 1.00 and 4.06 h post-dose (oral cohort) and 3.00 and 3.07 h post-start of infusion, i.e., at the end of infusion (IV cohort); declined in a biphasic manner and remained quantifiable between 120 and 336 h post-dose (oral cohort) and 147 and 339 h post-start of infusion (IV cohort). Whole-blood concentrations remained quantifiable between 96 and 240 h post-dose (oral cohort) and 123 and 339 h post-start of infusion (IV cohort; Fig. 3). In oral vs IV cohorts, the GM T_1/2_ was 61.6 vs 69.5 h; C_max_ was 9,740 vs 12,300 ng equiv/mL, AUC_(0-last)_ was 410,000 vs 562,000 ng equiv.h/mL, and AUC_(0-inf)_ was 446,000 vs 603,000 ng equiv.h/mL. Vd and CL were 99.6 vs 105 L and 18.7 vs 17.5 mL/min, respectively (Table 2). The arithmetic mean whole blood to plasma total radioactivity concentration ratios ranged from 0.684 to 0.928 for the oral cohort and 0.660 to 0.905 for the IV cohort.

### PK analysis: MGX and FMGX

**Fig 4:**
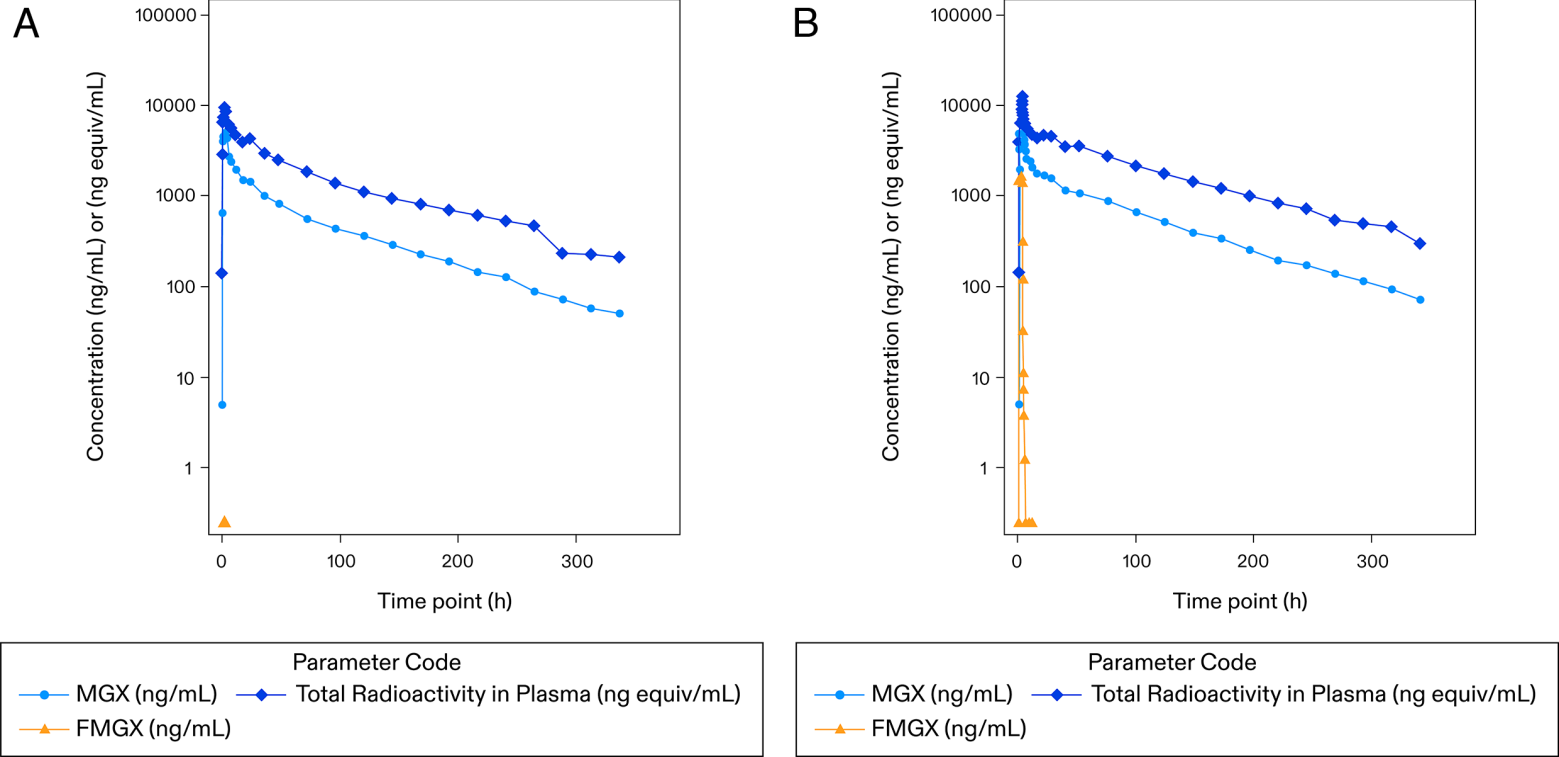
Geometric mean (×/÷ SD) MGX, FMGX, and total radioactivity plasma PK concentrations: Log10/linear scale (PK population). (**A**) Oral cohort: FMGX 500 mg + ^14^C NMT 3.1 MBq (84.0 µCi). (**B**) IV Cohort: FMGX 600 mg + ^14^C NMT 3.4 MBq (93.0 µCi) ^14^C, carbon-14; μCi, microcurie; FMGX, fosmanogepix; h, hour; IV, intravenous; MBq, megabecquerel; MGX, manogepix; NMT, not more than; PK, pharmacokinetics.

In both cohorts, MGX plasma concentrations were observed from 0.50 h in all participants. Maximum concentrations occurred between 1.00 and 3.00 h post-dose (oral cohort) and 3.01 and 3.17 h post-start of infusion, i.e., at the end of infusion (IV cohort), declined in a biphasic manner, and remained quantifiable between 168 and 336 h post-dose (oral cohort) and 243 and 339 h post-start of infusion (IV cohort; Fig. 4). In oral vs IV cohort, the GM MGX T_1/2_ was 60.0 vs 61.9 h; C_max_ was 5,620 vs 6,170 ng/mL, AUC_(0-last)_ was 151,000 vs 194,000 ng.h/mL, and AUC_(0-inf)_ was 159,000 vs 201,000 ng.h/mL. Vd and CL were 208 vs 215 L and 40.0 vs 40.1 mL/min, respectively. A comparison of GM C_max_/D, AUC_(0-last)_/D, and AUC_(0-inf)_/D, for the oral vs IV formulation, gave approximate values of 116%, 98.5%, and 100%, respectively. GM MPR C_max_ and MPR AUC_(0–last)_ were 4.30 and 58.5, respectively (Table 3). FMGX plasma concentrations (IV cohort) were observed from 0.50 h in all participants, maximum concentrations occurred between 0.50 and 3.07 h post-start of infusion and were quantifiable between 4 and 5 h post-start of infusion. The GM C_max_ was 1,880 ng/mL, and AUC_(0–last)_ was 4330 ng.h/mL.

**TABLE 3:** Summary of MGX plasma PK parameters (PK population)^*e*^

PK parameters^*a*^	Oral cohort [FMGX 500 mg + ^14^C NMT 3.1 MBq (84.0 µCi)] *n* = 5	IV cohort [FMGX 600 mg + ^14^C NMT 3.4 MBq (93.0 µCi)] *n* = 5
T_max_ (h)	1.52 (1.00–3.00)	3.07 (3.01–3.17)
C_max_ (ng/mL)	5,620 (26.4)	6,170 (10.6)
C_max_/D (ng/mL/mg)	14.7 (26.4)	12.7 (11.5)
AUC_(0–24)_ (ng.h/mL)	54,200 (33.0)	57,200 (18.2)
AUC_(0–last)_ (ng.h/mL)	151,000 (18.2)	194,000 (22.2)
AUC_(0–last)_/D (ng.h/mL/mg)	394 (18.2)	400 (20.6)
AUC_(0–inf)_ (ng.h/mL)	159,000 (17.0)	201,000 (24.5)
AUC_(0–inf)_/D (ng.h/mL/mg)	416 (17.0)	415 (22.8)
T_1/2_ (h)	60.0 (63.2)	61.9 (47.8)
CL^*b*^ (mL/min)	40.0 (17.0)	40.1 (22.8)
V_d_^*b*^ (L)	208 (61.2)	215 (35.7)
V_ss_ (L)	–^*f*^	200 (35.7)
MRT_(0–last)_^*c*^ (h)	61.5 (53.2)	72.7 (38.1)
MRT_(0–inf)_^*c*^ (h)	74.7 (67.1)	83.1 (47.5)
MPR C_max_^*d*^	–	4.30 (37.0)
MPR AUC_(0–last)_^*d*^	–	58.5 (31.7)

^*a*^ Reported as geometric mean (geometric coefficient of variation%) except for T_max_, which are reported as median (range).

^*b*^ CL/F and Vd/F for oral cohort.

^*c*^ Include MRT_(0–inf)__ev and MRT_(0–last)__ev for oral cohort and MRT_(0–inf)__iv and MRT_(0–last)__iv for IV cohort.

^*d*^ Presented for IV cohort only.

^*e*^ ^14^C, carbon-14; μCi, microcurie; AUC_(0–24),_ area under the curve (AUC) from 0 time to 24 h post-dose; AUC_(0–inf)_/D, AUC from 0 time extrapolated to infinity divided by actual dose; AUC_(0–inf),_ AUC from 0 time extrapolated to infinity; AUC_(0–last)_/D, AUC from 0 time to the last measurable concentration divided by actual dose; AUC_(0–last)_, AUC from 0 time to the last measurable concentration; CL, total body clearance after IV administration; C_max_/D, maximum observed concentration divided by actual dose; C_max,_ maximum observed concentration; FMGX, fosmanogepix; h, hour; IV, intravenous; MBq, megabecquerel; MPR C_max_, metabolite-to-parent ratio based on C_max;_ MPR AUC_(0–last),_ metabolite-to-parent ratio based on AUC_(0–last)_; MRT_(0–inf)__ev, mean residence time (MRT) extrapolated to infinity after extravascular administration; MRT_(0–inf)__iv, MRT extrapolated to infinity after IV administration; MRT_(0–last)__ev, MRT from 0 time to the last measurable concentration after extravascular administration; MRT_(0–last)__iv, MRT from 0 time to the last measurable concentration after IV administration; NMT, not more than; PK, pharmacokinetics; T_1/2,_ apparent elimination half-life; CL/F, total body clearance after extravascular administration; T_max,_ time of maximum observed concentration; Vd/F, apparent volume of distribution based on the terminal phase after extravascular administration; V_d,_ apparent volume of distribution based on the terminal phase after IV administration; V_ss,_ predicted volume of distribution at steady state after IV administration.

^*f*^ –, not calculated.

### Metabolite profiling and structural identification

**Fig 5:**
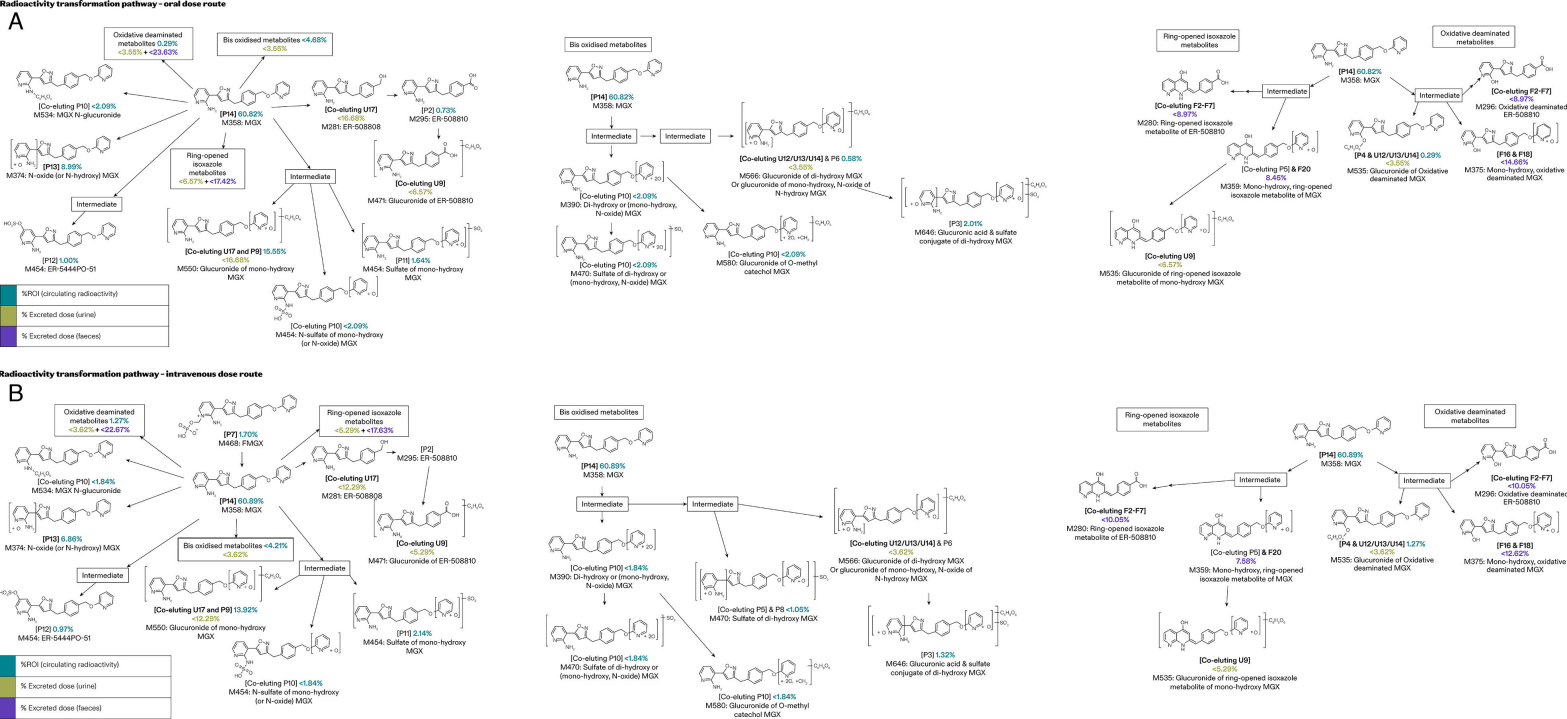
Radioactive transformation pathways. (**A**) Oral dose route. (**B**) IV dose route. FMGX, fosmanogepix; IV, intravenous; MGX, manogepix; ROI, Region of Interest.

The radioactivity transformation pathways for both oral and IV dose routes indicated multiple major routes of metabolism and elimination for FMGX (Fig. 5), with the main routes of metabolism via the formation of MGX followed by oxidation, oxidative deamination, and conjugation (e.g., with glucuronic acid or sulfate; Table 4). Unusual metabolites, believed to be formed by isoxazole ring-opening and further metabolism, were observed. All the key human plasma metabolites were observed in the toxicity species, except for the sulfate of di-hydroxy (or mono-hydroxy, N-oxide) MGX (Co-eluting P10), which was not observed in rat plasma. Semi-quantification indicated that for the majority of key human metabolites, the approximate concentrations were greater in at least one toxicity species compared with human, except for sulfate of di-hydroxy (or mono-hydroxy, N-oxide) MGX (Co-eluting P10), and di-hydroxy (or mono-hydroxy, N-oxide) MGX (Co-eluting P10). However, since these metabolites represented <10% of the total drug-related material in the human AUC plasma samples, these were not considered to be of toxicological concern as per regulatory guidance on metabolite safety testing (13).

**TABLE 4:** Summary of metabolites identified^*c*^^,^^*e*^

Metabolite(s)	Metabolic pathway	Oral cohort [FMGX 500 mg + ^14^C NMT 3.1 MBq (84.0 µCi)] *n* = 5			IV cohort [FMGX 600 mg + ^14^C NMT 3.4 MBq (93.0 µCi)] *n* = 5		
		Plasma	Urine	Feces	Plasma	Urine	Feces
M280	Ring-opened isoxazole metabolite of ER-508810	–^*f*^	–	F2-F7^*a*^	–	–	F2-F7^*a*^
M296	Oxidative deaminated ER-508810	–	–	F2-F7^*a*^	–	–	F2-F7^*a*^
M295	ER-508810	P2	–	–	*^b^(P2)*	–	–
M471	Glucuronide of ER-508810	–	U9^*a*^	–	–	U9^*a*^	–
M535	Glucuronide of ring-opened isoxazole metabolite of mono-hydroxy MGX	–	U9^*a*^	–	–	U9^*a*^	–
M646	Glucuronide & sulfate conjugate of di-hydroxy MGX	P3	–	–	P3	–	–
M566	Glucuronide of mono-hydroxy, N-oxide (or N-hydroxy) MGX	–	U13-U14	–	–	U12-U13^*a*^	–
M535	Glucuronide of oxidative deaminated MGX	P4	U13-U14	–	P4	U12-U14^*a*^	–
M359	Mono-hydroxy ring-opened isoxazole metabolite of MGX	*^b^(P5)*	–	–	P5	–	–
M470	Sulfate of di-hydroxy MGX	*^b^(P5)*	–	–	P5	–	–
M375	Mono-hydroxy, oxidative deaminated MGX	–	–	F16^*a*^	–	–	F16^*a*^
M566	Glucuronide of di-hydroxy MGX	P6	–	–	*^b^(P6)*	–	–
M468	FMGX	*^b^(P7)*	–	–	P7^*a*^	–	–
M470	Sulfate of di-hydroxy MGX	*^b^(P8)*	–	–	P8	–	–
M281	ER-508808	–	U17^*a*^	–	–	U17^*a*^	–
M550	Glucuronide of mono-hydroxy MGX	P9^*a*^	U17^*a*^	–	P9^*a*^	U17^*a*,*d*^	–
M375	Mono-hydroxy, oxidative deaminated MGX (2nd isomer)	–	–	F18^*a*^	–	–	F18^*a*^
M359	Ring-opened isoxazole metabolite of mono-hydroxy MGX	–	–	F20^*a*^	–	–	F20^*a*^
M534	MGX N-glucuronide	P10	–	–	P10	–	–
M454	N-sulfate of mono-hydroxy (or N-oxide) MGX	P10	–	–	P10	–	–
M470	Sulfate of di-hydroxy (or mono-hydroxy, N-oxide) MGX	P10	–	–	P10	–	–
M390	Di-hydroxy (or mono-hydroxy, N-oxide) MGX	P10	–	–	P10	–	–
M580	Glucuronide of O-methyl catechol MGX	P10	–	–	P10	–	–
M454	Sulfate of mono-hydroxy MGX	P11	–	–	P11	–	–
M454	ER-5444PO-51	P12	–	–	P12	–	–
M374	N-oxide (or N-hydroxy) MGX	P13^*a*^	–	–	P13^*a*^	–	–
M358	MGX	P14^*a*^	–	–	P14^*a*^	–	–

^*a*^ Indicates >5% circulating radioactivity in at least one plasma sample or >5% of the dose in at least one urine or feces sample; where the same region identifier is assigned to more than one component, these correspond to co-eluting metabolites present in the sample.

^*b*^ Present by LC-MS but does not correspond to an assigned region of interest (less than 2× background peak height in the radiochromatogram for samples). Italicized bracketed assignment indicates the metabolite number based on retention time and correlation with other samples (oral and intravenous dose route).

^*c*^ Component below threshold required for metabolite identification.

^*d*^ Based on LC-MS response, the major component under U17 is M550: glucuronide of mono‑hydroxy MGX. FMGX, fosmanogepix; LC-MS, liquid chromatography tandem mass spectrometry; MGX, manogepix.

^*e*^ Assigned components corresponding to notable metabolites designated as, for example, “P1,” where: first character indicates the matrix (“P/U/F” = plasma/urine/feces) followed by a sequential number.

^*f*^ –, not detected.

### Safety

No deaths, serious AEs, or severe AEs were reported, and no participant was withdrawn due to an AE. One (10.0%) participant in the IV cohort reported one mild AE of headache, which was considered unrelated to FMGX by the PI. The AE was resolved before the end of the study and did not require treatment. No clinically significant findings in any laboratory assessments, vital signs, electrocardiograms (ECGs), or physical examinations were observed.

## DISCUSSION

FMGX, a novel antifungal, is currently under clinical development for the treatment of various IFDs (6). This Phase 1, single-dose clinical study in healthy male participants was the first to evaluate the disposition and metabolism of FMGX. It provided data on routes of elimination, distribution, and the metabolic profile of FMGX along with identification of its key metabolites that potentially augment the safety and PK data provided in previous Phase 1 clinical studies.

In this study, participants were enrolled into the oral cohort (^14^C-FMGX 500 mg) and the IV cohort [^14^C- FMGX 600 mg (via a 3-h infusion)]. In the oral cohort, 90.2% of the radioactivity administered was recovered over the full sampling period of 456 h (82.3% within 240 h), with 46.4% recovered in urine and 43.8% recovered from feces, suggesting an almost equal split of renal and hepatic clearance in excretion of FMGX-related metabolites. Additionally, only 4% of drug-related material was eliminated in the feces within the first 48 h post-dose period, suggesting extensive metabolism and absorption of MGX. Similar findings were reported in healthy participants receiving oral [cyano-^14^C] isavuconazonium sulfate, an antifungal prodrug. Over the 600-h study period, a mean of 91.6% of the radioactivity administered was recovered, with 45.5% recovered in urine and 46.1% in feces (14). In the IV cohort, 82.4% of the radioactivity administered was recovered (76.2% within 264 h), with 42.5% recovered in urine and 39.9% recovered from feces, suggesting an almost equal split of renal and hepatic clearance in excretion of FMGX-related metabolites. The substantial amount of total radioactivity recovered in the feces confirmed the elimination of FMGX-related materials in bile. Similar results were observed in healthy participants after receiving an IV dose of rezafungin, an antifungal drug. In the first 17 days of the study (384 h), 52% of the cumulative recovery of radioactivity was collected from excreta, including 38% in feces, suggesting biliary excretion, intestinal secretion, and passive diffusion (15). The findings in our study also extend the observation reported in a pre-clinical absorption, distribution, metabolism and elimination (ADME) study in cynomolgus monkeys. Administration of ^14^C-FMGX in monkeys (6 mg/kg IV) demonstrated an overall radioactivity recovery of 87.6%, with 49.8% recovered in feces and 20.6% in urine (9). The comparable rates and routes of elimination for the total radioactivity, between the oral and IV administration routes in our study, further support the evidence of extensive absorption of the oral dose with minimal pass through.

In both cohorts, MGX formation was rapid, with plasma concentrations quantifiable from the first sampling point of 0.5 h. The median T_max_ was 1.52 h in the oral cohort and coincided with the end of infusion at 3.07 h in the IV cohort. This was similar to findings in a previous Phase 1 study of FMGX in healthy participants (4). The median T_max_ (3.0 h) for the single- and multiple-dose regimens was concordant with the 3-h infusion time (4). Exposure to MGX accounted for only 44% and 36% of circulating plasma total radioactivity based on AUC_(0–24)_ and AUC_(0–inf)_, respectively, suggesting that there were additional circulating components in plasma not quantified following ^14^C-FMGX administration. Similar findings were reported in a study of oral isavuconazonium sulfate in healthy participants. In plasma, mean radioactivity (cyano-^14^C) concentrations were higher compared to that of isavuconazole, indicating the presence of additional isavuconazole metabolites (14). Comparison (oral vs IV formulation) of GM MGX C_max_/D (~116%), AUC_(0–last)_/D (~98.5%), and AUC_(0–inf)_/D (~100%) and total radioactivity C_max_/D (~101%), AUC_(0–last)_/D (~92.4%), and AUC_(0–inf)_/D (~93.8) demonstrated almost complete bioavailability after oral administration. These findings extend the observations in previous Phase 1 oral and IV FMGX studies in healthy volunteers, where absolute bioavailability of MGX, estimated as the dose-corrected ratio of the oral-to-IV GM AUC _(0–inf)_ ranged from 90.6% to 101.2%, indicating complete absorption of FMGX and conversion to MGX (4).

The volume of distribution [208 L (oral cohort); 215 L (IV cohort)] observed was higher than total body water indicating distribution into tissue. In addition, the volume of distribution of 208 L for the oral dose, compared to 215 L for the IV infusion, further supports the extensive bioavailability of the oral formulation. The geometric mean clearance value (40 mL/min) and the estimated plasma half-life (60–62 h) were similar following oral doses and IV infusions, indicating that the rate of drug elimination from the body and the time taken for elimination were comparable for both routes of administration.

The radioactivity transformation pathways indicated multiple major routes of metabolism and elimination for FMGX. The main routes of metabolism were via MGX and included oxidation, oxidative deamination, and conjugation (e.g., conjugation with glucuronic acid or sulfate). Only two metabolites represented 10% or more of the total circulating radioactivity in one or more participants: glucuronide of mono-hydroxy MGX (P9) and N-oxide (or N-hydroxy) MGX (P13). Previous metabolite identification studies were conducted in rats and monkeys. In both these animal models, co-administration of CYP3A4 inhibitors was not required to achieve target antifungal concentrations (9). The key human plasma metabolite not observed in rat plasma, sulfate of di-hydroxy (or mono-hydroxy, N-oxide) MGX (co-eluting P10), represented <10% of the total drug-related material in the human AUC plasma samples. Therefore, as per regulatory guidance on metabolite safety testing (13), this metabolite was not considered to be of toxicological concern.

No new safety signals were detected during the study. There were no clinically significant abnormalities in any laboratory assessments, vital signs, ECGs, or physical examinations. These findings are concordant with the safety results reported in previous Phase 1 and Phase 2 clinical studies of FMGX (4, 5).

In conclusion, MGX was found to be extensively metabolized, with only two metabolites in at least one participant accounting for >10% of total circulating radioactivity. In addition, all key human plasma metabolites were also identified in preclinical animal studies (no unique human metabolites were identified). The elimination profile of FMGX (equally through renal and hepatic routes) indicates that dosage adjustments may be required in patients with hepatic or renal insufficiency. These findings provide important information about the PK, metabolism, and elimination of FMGX that may guide its development for further clinical use.

## Data Availability

Upon request, and subject to review, Pfizer will provide the data that support the findings of this study. Subject to certain criteria, conditions, and exceptions, Pfizer may also provide access to the related individual de-identified participant data. See https://www.pfizer.com/science/clinical-trials/trial-data-and-results for more information.

## References

[1] Chakrabarti A, Mohamed N, Capparella MR, Townsend A, Sung AH, Yura R, Muñoz P. 2022. The role of diagnostics-driven antifungal stewardship in the management of invasive fungal infections: a systematic literature review. Open Forum Infect Dis 9:ofac234. doi:10.1093/ofid/ofac234 35873300 PMC9297315

[2] Bajpai VK, Khan I, Shukla S, Kumar P, Rather IA, Park Y-H, Huh YS, Han Y-K. 2019. Invasive fungal infections and their epidemiology: measures in the clinical scenario. Biotechnol Bioproc Eng 24:436–444. doi:10.1007/s12257-018-0477-0

[3] Zhen C, Lu H, Jiang Y. 2022. Novel promising antifungal target proteins for conquering invasive fungal infections. Front Microbiol 13:911322. doi:10.3389/fmicb.2022.911322 35783432 PMC9243655

[4] Hodges MR, Ople E, Wedel P, Shaw KJ, Jakate A, Kramer WG, Marle S van, van Hoogdalem E-J, Tawadrous M. 2023. Safety and pharmacokinetics of intravenous and oral fosmanogepix, a first-in-class antifungal agent, in healthy volunteers. Antimicrob Agents Chemother 67:e0162322. doi:10.1128/aac.01623-22 36988461 PMC10112065

[5] Vazquez JA, Pappas PG, Boffard K, Paruk F, Bien PA, Tawadrous M, Ople E, Wedel P, Oborska I, Hodges MR. 2023. Clinical efficacy and safety of a novel antifungal, fosmanogepix, in patients with candidemia caused by Candida auris: results from a phase 2 trial. Antimicrob Agents Chemother 67:e0141922. doi:10.1128/aac.01419-22 37022196 PMC10190264

[6] Shaw KJ, Ibrahim AS. 2020. Fosmanogepix: a review of the first-in-class broad spectrum agent for the treatment of invasive fungal infections. J Fungi (Basel) 6:239. doi:10.3390/jof6040239 33105672 PMC7711534

[7] Hoenigl M, Sprute R, Arastehfar A, Perfect JR, Lass-Flörl C, Bellmann R, Prattes J, Thompson GR, Wiederhold NP, Al Obaidi MM, Willinger B, Arendrup MC, Koehler P, Oliverio M, Egger M, Schwartz IS, Cornely OA, Pappas PG, Krause R. 2022. Invasive candidiasis: investigational drugs in the clinical development pipeline and mechanisms of action. Expert Opin Investig Drugs 31:795–812. doi:10.1080/13543784.2022.2086120 PMC9339492 35657026

[8] Jacobs SE, Zagaliotis P, Walsh TJ. 2021. Novel antifungal agents in clinical trials. F1000Res 10:507. doi:10.12688/f1000research.28327.2 35136573 PMC8787557

[9] Mansbach R, Shaw KJ, Hodges MR, Coleman S, Fitzsimmons ME. 2017. Absorption, distribution, and excretion of ^14^C-APX001 after single-dose administration to rats and monkeys. Open Forum Infect Dis 4:S472. doi:10.1093/ofid/ofx163.1209

[10] Coppola P, Andersson A, Cole S. 2019. The importance of the human mass balance study in regulatory submissions. CPT Pharmacometrics Syst Pharmacol 8:792–804. doi:10.1002/psp4.12466 31515957 PMC6916658

[11] Penner N, Xu L, Prakash C. 2012. Radiolabeled absorption, distribution, metabolism, and excretion studies in drug development: why, when, and how? Chem Res Toxicol 25:513–531. doi:10.1021/tx300050f 22309195

[12] ClinicalTrials.gov. 2022. A study to assess the mass balance recovery and metabolite profile & identification of [^14^C]-APX001 in healthy males. Available from: https://clinicaltrials.gov/ct2/show/NCT04804059. Retrieved 01 May 2022.

[13] Services USDoHaH, Administration FaD, (CDER) CfDEaR. 2020. Safety testing of drug metabolites guidance for industry. Administration FaD. Available from: https://www.fda.gov/media/72279/download

[14] Townsend R, Kato K, Hale C, Kowalski D, Lademacher C, Yamazaki T, Akhtar S, Desai A. 2018. Two phase 1, open‐label, mass balance studies to determine the pharmacokinetics of ^14^C‐labeled Isavuconazonium sulfate in healthy male volunteers. Clin Pharmacol Drug Dev 7:207–216. doi:10.1002/cpdd.376 28750160 PMC5811773

[15] Ong V, Wills S, Watson D, Sandison T, Flanagan S. 2022. Metabolism, excretion, and mass balance of [^14^C]-rezafungin in animals and humans. Antimicrob Agents Chemother 66:e0139021. doi:10.1128/AAC.01390-21 34662192 PMC8765310

